# Targeting the BMI1-Noxa axis by Dioscin induces apoptosis in oral squamous cell carcinoma cells

**DOI:** 10.7150/jca.100631

**Published:** 2025-01-01

**Authors:** Jinglin Fang, Ruirui Wang, Xiaoying Li, XiaoCong Wang, Lingling Gong, An Lou, Jie Lu, Xiaoyue Zhang, Qin Zhao, Xinfang Yu, Qiling Zhou, Ming Li

**Affiliations:** 1 School of Stomatology, Hunan University of Chinese Medicine, Changsha, Hunan 410208, China.; 2 Changsha Stomatological Hospital, Changsha, Hunan 410004, China.; 3 Department of Radiology, The Third Xiangya Hospital of Central South University, Changsha, Hunan 410013, China.; 4 Changsha Health Vocational College. Changsha, Hunan 410600, China.; 5 Key Laboratory of Carcinogenesis and Cancer Invasion of Chinese Ministry of Education, XiangYa Hospital, Central South University, Changsha, Hunan 410008, China.; 6 Key Laboratory of Carcinogenesis of National Health Commission, Cancer Research Institute and School of Basic Medical Science, Xiangya School of Medicine, Central South University, Changsha, Hunan 410008, China.

**Keywords:** Oral squamous cell carcinoma, Noxa, BMI1, Dioscin

## Abstract

Dioscin is a natural plant-derived steroidal saponin that exerts antitumor effects in multiple cancers. It is widely involved in multiple apoptotic pathways and exerts its anti-tumor effects. In this study, we discovered that Dioscin treatment increased the expression of Noxa, thereby inducing the apoptosis of OSCC cells. Previous reports indicate that dysfunction of the BMI1-Noxa axis is frequently observed in multiple cancers. Our study revealed that Dioscin upregulates Noxa by impairing the protein expression of BMI1 in OSCC cells. Dioscin promotes the ubiquitination of BMI1 and facilitates its degradation, leading to upregulation of Noxa expression at the mRNA level and activation of apoptosis. Additionally, Dioscin exhibited potent tumor suppression in xenograft tumor models. In conclusion, our research provides new insights and strategies for inhibiting OSCC cells by investigating the ant-tumor mechanism of the natural compound Dioscin.

## Introduction

Oral squamous cell carcinoma (OSCC) is the most common subtype of head and neck squamous cell carcinoma (HNSCC). According to data from the Global Cancer Observatory (GCO), 377,713 instances of OSCC were identified globally in 2020. It is predicted that by 2040, the incidence of OSCC will rise by around 40%, accompanied by an increase in mortality^1^. The pathogenesis of OSCC is complex, involving abnormal activation of oncogenic signaling, aberrant inactivation of inhibitory signaling, DNA methylation, and dysregulation of the tumor microenvironment, all potentially contributing to tumorigenesis^2^. Therefore, continued research into the mechanisms driving OSCC development is crucial. Finding effective treatments that can mitigate the rising incidence and mortality rates while minimizing side effects is essential for improving outcomes and quality of life for OSCC patients.

Noxa is a pro-apoptotic member of the Bcl-2 family and belongs to the BH3-only protein subclass. While the pro-apoptotic effect of Noxa on tumors may be relatively weak in isolation, it is a critical player in the interaction with several proteins in the apoptotic pathway^3^. One of the most widely recognized effects of Noxa is its selective inhibition of Mcl-1 expression^4^, which is overexpressed in many cancers, leading to chemoresistance and poor prognoses for cancer patients^5^. Furthermore, the Noxa gene and protein regulation is intricate, as they can be activated by a wide range of signals related to cellular stress, DNA damage, and cell division and proliferation^6^. Early observations suggested that Noxa transcription is primarily induced by p53^7^, but a growing body of research now indicates that it can also be activated in a p53-independent manner. For example, hypoxia-inducible factor 1-α (HIF-1α) induces Noxa expression through the hypoxia-responsive element of the Noxa promoter, resulting in cell death brought on by hypoxia^8^. Oncogenic stress can induce Noxa expression in a p53-independent manner^9^. Overexpressing adenovirus E1A protein in SH-SY5Y neuroblastoma cells (which lack functional p53) and SaOS-2 cells activates p73 and induces the expression of Noxa mRNA^10^. However, the pathway through which Noxa is induced in oral squamous cell carcinoma (OSCC) still requires further investigation.

B cell-specific Moloney murine leukemia virus integration site 1 (BMI1) is an intracellular oncoprotein characterized by three significant structural domains: an N-terminal RING finger (RF) domain, a C-terminal Proline-Serine-rich (PS/PEST) domain, and a central helix-turn-helix (HTH) domain^11^. The N-terminal RING finger domain and the C-terminal Proline-Serine-rich domain play crucial roles in the transcriptional activation and immortalization of telomerase reverse transcriptase (TERT) in epithelial cells^12^. Additionally, the N-terminal RING finger domain functions as a recognition motif for the F-box protein β-TrCP (containing β-transducin repeats), which controls the ubiquitination and proteasome-mediated degradation of various proteins^13^. BMI1 is critical in regulating the cell cycle, cellular immortalization and senescence^14^, ^15^. BMI1 is linked to the initiation and progression of a variety of tumor-initiating cells, playing an important role in the development and progression of cancer^16^-^18^. BMI1 exhibits a consistent mode of action across various cancers, including ovarian cancer, nasopharyngeal carcinoma, and non-small cell lung cancer^12^, ^19^-^22^. Targeting BMI1 offers a promising direction for tumor treatment.

Dioscin (Diosin, DIO) is a natural steroidal saponin found abundantly in medicinal plants belonging to Dioscoreaceae, Liliaceae, Sphagnum, and Rosaceae families^23^. It is a major bioactive compound in Dioscorea nipponica Makino^24^, ^25^. Early researches have indicated that Dioscin can produce steroidal hormone to exert pharmacological effects such as antibacterial, anti-inflammatory, anti-allergic, anti-viral, anti-shock, and hypolipidemic. Consequently, Dioscin has been extensively employed in the treatment of cardiovascular diseases such as hypertrophy, arrhythmias, angina pectoris, and coronary artery disease^26^, as well as metabolic disorders including non-alcoholic fatty liver disease, diabetes mellitus, hyperuricemia, and osteoporosis^27^-^30^. Multiple studies have indicated that Dioscin is also beneficial in anti-tumor treatment, with different and complex mechanisms such as DNA damage induction and cell cycle arrest, apoptosis induction, EMT suppression, and macrophage polarization regulation. Dioscin inhibits colorectal cancer via regulating macrophage polarization and MDSC differentiation^31^. Dioscin suppresses TGF-β1-inhibited EMT, lowers lung cancer cell invasion, inhibits MEK/ERK and PI3K/AKT signaling pathways, and overcomes treatment resistance^32^. Many medications containing Diosin are currently in use in the clinic, including "Di-Ao-Xin-Xue-Kang" capsules for the treatment of coronary heart disease, atherosclerosis, and other cardiovascular diseases^33^, and Dioscorea bulbifera for the treatment of goiter^34^. In conclusion, Dioscin is an effective but non-toxic medicine that targets infected or sick tissues while sparing normal tissues, making it a viable multi-target therapeutic option for treating a variety of disorders. Previous research has demonstrated that Diosicn inhibits survivin expression and induces apoptosis by interrupting the binding of EGFR to AT-rich sequences at the survivin promoter^35^. However it remains unclear whether Dioscin can also target additional signaling pathways or modulate target molecules through protein modifications to enhance its antitumor effects. In this study, we demonstrate that Dioscin targets the BMI1-Noxa axis and facilitates BMI1 degradation by ubiquitination to exert its anti-tumor activity.

## Materials and Methods

### Reagents and plasmid constructs

Dioscin (#HY-N0124, purity>99%) and Z-VAD-FMK (#HY-16658) were purchased from MCE. Necrostatin-1 (#S8037) was purchased from Selleckchem. Cycloheximide (CHX), MG132 and transfection reagent LipofectamineTM 2000 (#11668019) were purchased from Thermo Fisher Scientific. Antibodies against β-actin (#4970), α-Tubulin (#3873), BMI1 (#6964), ubiquitin (#3936), Noxa (#3724), β-TrCP (#4394S), Bax (#5023), cytochrome C (#11940), cleaved-caspase3 (#9664), and VDAC1 (#4661) were bought from Cell Signaling Technology. Luciferase reporter gene pGL3-Noxa-N1 (#26112) is available from Addgene.

### Cell lines and cell culture

The OSCC cells include CAL27, SCC9 and SCC25. CAL27 is an epithelial cell collected from diseased tissue in the middle of the tongue of a 56-year-old white male. SCC9 was collected from the tongue of a 25-year-old man with squamous cell carcinoma. SCC25 was collected from the tongue of a 70-year-old male with squamous cell carcinoma. 293T is epithelial-like cells collected from the kidneys of patients. All cells were bought from the American Typical Culture Collection. CAL27, SCC9 and SCC25 were cultured in DMEM/F12 medium with 10% FBS and 1% antibiotics. 293T cells were maintained in DMEM medium with 10% FBS. All cells were incubated at 37 ℃ in a humidified atmosphere with 5% CO_2_. Prior to freezing, the cells underwent cytogenetic examination and documentation. Each vial of frozen cells was thawed and stored for two months (10 passes), after which the cells were cultured for 36-48 hours before their proteins were extracted for analysis.

### MTS assay

The MTS assay was performed as formerly depicted^36^. Briefly, The OSCC cells were seeded in 96-well plates (4×10^3^ cells/well) and incubated overnight to allow for cell adhesion. The cells were then treated with varied Dioscin concentrations for 24, 48, and 72 hours. Finally, cell viability was measured by assessing the optical density of each well at 450 nm after adding the MTS reagents (#G3580 Promega).

### Soft agar assay

The soft agar assay was performed as formerly depicted^37^. Briefly, the OSCC cells were then seeded in 6-well plates (8×10^3^ cells/well) containing basal DMEM-F12 media with 0.6% agar and 10% FBS, along with various concentrations of Diosicn. After incubating for 14 days at 37 ℃ in a CO_2_ incubator, the colonies were examined and counted.

### Immunofluorescence (IF)

The Immunofluorescence (IF) was performed as formerly depicted^38^. Briefly, the OSCC cells were exposed to various doses of Dioscin for 48 hours. After removing the culture medium, cells were washed on a shaker for five minutes each. The cells were then fixed with 4% paraformaldehyde for 10 minutes, followed by permeabilization with 0.3% Triton X-100 for 15 minutes. Subsequent washing with PBS as previously described. The cells were blocked with 10% BSA solution and incubated overnight at 4 ℃ with primary antibodies. The following day, cells were incubated with corresponding fluorescent secondary antibodies at room temperature for 30 minutes. Finally, the nuclei were labeled with DAPI and examined using fluorescence microscopy.

### Western blotting (WB) assay

WB was implemented as formerly depicted^39^. Briefly, the OSCC cells were exposed to different doses of Dioscin and then lysed using RIPA lysis buffer to obtain whole cell extracts (WCE). Protein content quantification was conducted utilizing the BCA Protein Assay Kit (#23228, Thermo Fisher Scientific), and then, equilibrating protein concentration. an aliquot of the protein sample was subjected to 10% SDS-PAGE gel and transferred onto a PVDF membrane. The membrane was blocked with 5% skimmed milk for 60 min, followed by incubation with a primary antibody at 4°C overnight. The membrane was then incubated with a secondary antibody for 60 min at room temperature. Finally, the Enhanced Chemiluminescence Reagent (ECL) (#34579, Thermo Fisher Scientific) was used to detect the target protein bands.

### qRT-PCR test

The qRT-PCR text was implemented as formerly depicted^38^. Total RNA was obtained from the OSCC cells treated with Dioscin using a TRIzol reagent. cDNA was subsequently synthesized using the PrimeScriptTM RT kit (#RR047A Takara Bio). qRT-PCR analysis (Tli RNaseH Plus) was performed with SYBR premix Ex Taq (#RR420A Takara Bio) according to the manufacturer's instructions. The relative amount of mRNA was normalized to GAPDH RNA, and expression levels were determined using the 2ΔΔCt method.

### Ubiquitination (Ub) analysis

Ub analysis was implemented as formerly depicted^40^. The OSCC cells were treated with different concentrations of Dioscin and co-incubated with MG132 to obtain whole-cell lysate. Subsequently, the cell lysate was disrupted using RIPA buffer supplemented with 0.1% SDS. The disruption involved sonication for 30 seconds, followed by boiling at 95 ℃ for 15 minutes. The mixture was then centrifuged at 12000×g for 15 minutes at 4 ℃. After collecting the supernatant, the protein concentration was measured. Anti-BMI1 antibody and agarose beads were added and incubated overnight at 4 ℃. WB analysis was performed to detect BMI1 ubiquitination the next day.

### Co-IP

The Co-IP was implemented as formerly depicted^41^. The OSCC cells were treated with or without Diosin and co-incubated with MG132. The cell lysates were produced using the IP buffer, and the protein concentration was determined with the BCA Protein Assay Kit. Equal amounts of lysates were flipped with the corresponding primary antibodies and protein A/G agarose beads at 4 ℃ overnight. The beads were washed with ice-cold PBS and then suspended in 40μl of 1×SDS-PAGE loading solution to obtain the supernatant. SDS-PAGE electrophoresis was performed to separate the target protein, which was then transferred onto the PVDF membrane. After blocking with 5% non-fat milk for 1 hour at 37 ℃, the membranes were exposed to the primary antibody overnight at 4 ℃. This was followed by incubation with the suitable secondary antibody for 1 hour at room temperature. The protein bands were detected using the enhanced chemiluminescence reagents.

### Cycloheximide (CHX) assay

The OSCC cells were pretreated with Dioscin for 48h, followed by treatment with CHX at various time points. Subsequently, whole cell lysates were collected to detect changes in BMI1 half-life using WB.

### Immunohistochemical (IHC)

IHC was implemented as formerly depicted^42^. Tissue slides from xenograft tumors were dewaxed in xylene, rehydrated using ethanol, and submerging in boiling sodium citrate buffer (10 mM, pH 6.0) for 10 minutes. The slides were then treated with 3% H₂O₂ for 10 minutes. Next, the slides were washed with phosphate-buffered saline (PBS), blocked with 10% goat serum albumin for 1 hour at room temperature and then incubated overnight at 4 ℃. Next day, incubate under room temperature conditions for 45 minutes with secondary antibodies. Target proteins were visualized using the DAB Substrate Kit (#34002; Thermo Fisher Scientific, Inc.).

### Xenograft mouse model

In compliance with the ethical requirements of the Medical Research Animal Ethics Committee of Central South University (Changsha, China), a total of 2 million CAL27 cells were injected subcutaneously into the right flank of 6-week-old athymic nude mice to establish a xenograft tumor model. Tumor volume was calculated using the formula: length multiplied by the square of the breadth, divided by 2. Measurements of tumor volume and mouse body weight were taken every two days. Once the tumor volume reached approximately 100 mm^3^, either a medium control (0.5% dimethyl sulfoxide in 100 μl corn oil/every 2 days) or Dioscin (10 mg/kg/in 100μl corn oil every 2 days) was given intraperitoneally. The mice were euthanized with CO_2_ (3L/min for 5 min) when the tumor volume reached approximately 800 mm^3^. Tumor tissues were then collected for weight measurement and IHC analysis. Additionally, serum was collected to analyze white blood cells (WBC), red blood cells(RBC), hemoglobin(Hb), and alanine aminotransferase(ALT), blood urea nitrogen(BUN), along with aspartate aminotransferase (AST) levels.

### Statistical analysis

The statistical significance was analyzed using the GraphPad Prism program. The data are presented as the mean ± standard deviation (SD) from at least three independent measurements. The statistical analysis of the data involved the utilization of Student's t-test and one-way ANOVA to compare the different groups. A significance criterion of p<0.05 was used to determine statistically significant differences.

## Results

### Dioscin inhibits OSCC tumor cell growth *in vitro*

**Figure 1:**
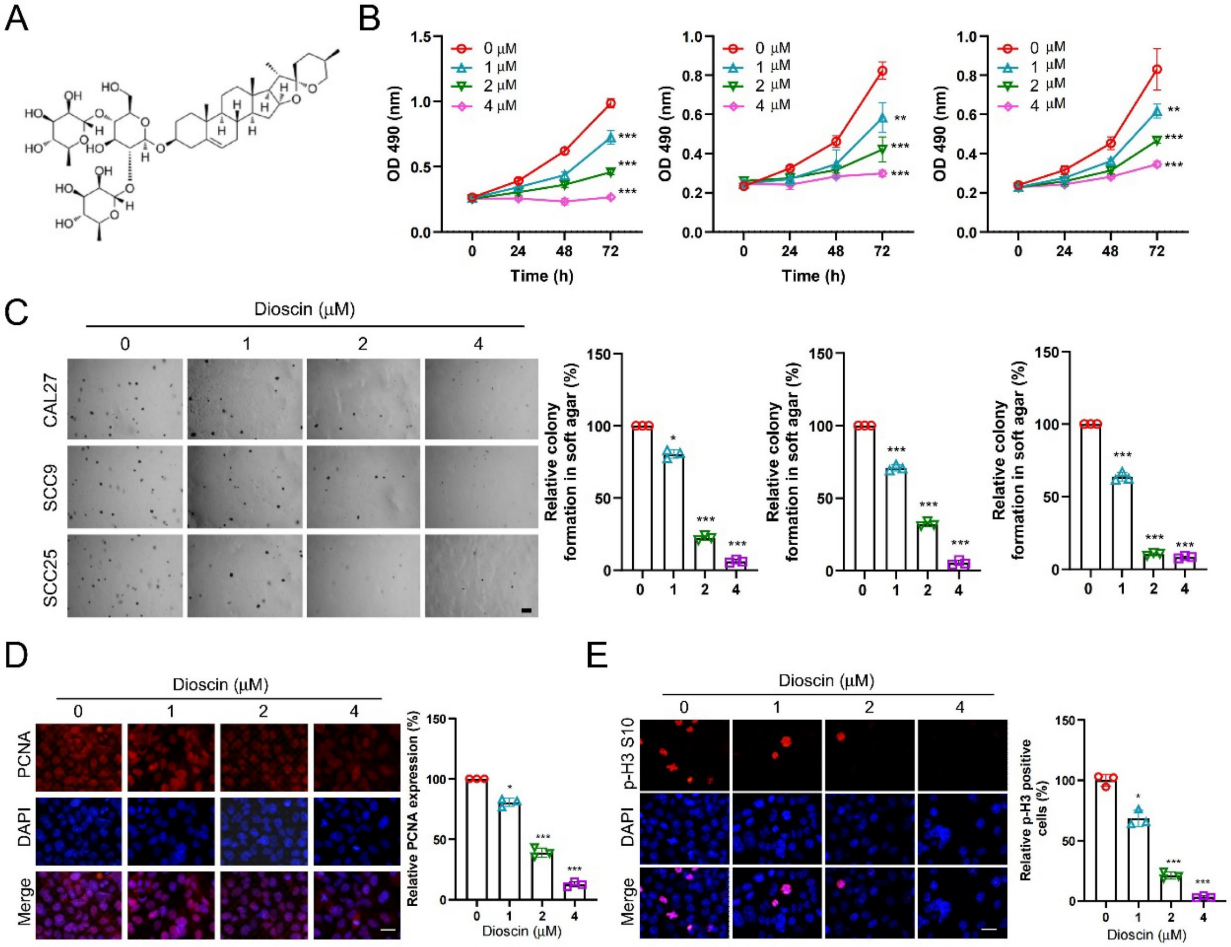
Dioscin inhibits the growth of OSCC tumor cells *in vitro*. A. Chemical structure of Dioscin. B. CAL27 (left), SCC9 (middle) and SCC25 (right) cells were treated with Dioscin (0/1/2/4 μM) for 24, 48, and 72 h. MTS was performed to determine the cell viability. C. CAL27 (top), SCC9 (middle) and SCC25 (bottom) cells were treated with Dioscin (0/1/2/4 μM) for 48 h, soft agar assay was used to detect anchorage-independent growth capacity. Scale bar, 500 μm. D and E. CAL27 cells were treated with Dioscin (0/1/2/4 μM) for 48 h, Immunofluorescence assay was used to detect PCNA and p-H3 S10 expression.

Dioscin (Figure 1A) has demonstrated antitumor activity against various human cancers. To characterize the antitumor effects of Dioscin in OSCC cells, we conducted a cell viability assay, and the MTS data showed that Dioscin significantly reduced the cell viability of CAL27, SCC9, and SCC25 cells in a time- and dose-dependent manner (Figure 1B). Meanwhile, the colony formation ability of CAL27, SCC9, and SCC25 cells was also significantly reduced dose-dependently using soft agar assay (Figure 1C). To further explore the effect of Dioscin on the proliferation of OSCC cells at the molecular level, we performed immunofluorescence on Dioscin-treated OSCC cells to detect the expression of tumor mitotic and proliferative markers p-H3 S10 and PNCA. The results showed a concentration-dependent reduction in p-H3 S10- and PCNA-positive cells in the experimental group compared to the control group (Figure 1D and Figure 1E). In conclusion, these findings suggest that Dioscin attenuates the viability, colony-forming ability, and proliferation of OSCC cells in a concentration-dependent manner.

### Dioscin induces intrinsic apoptosis in OSCC cells

**Figure 2:**
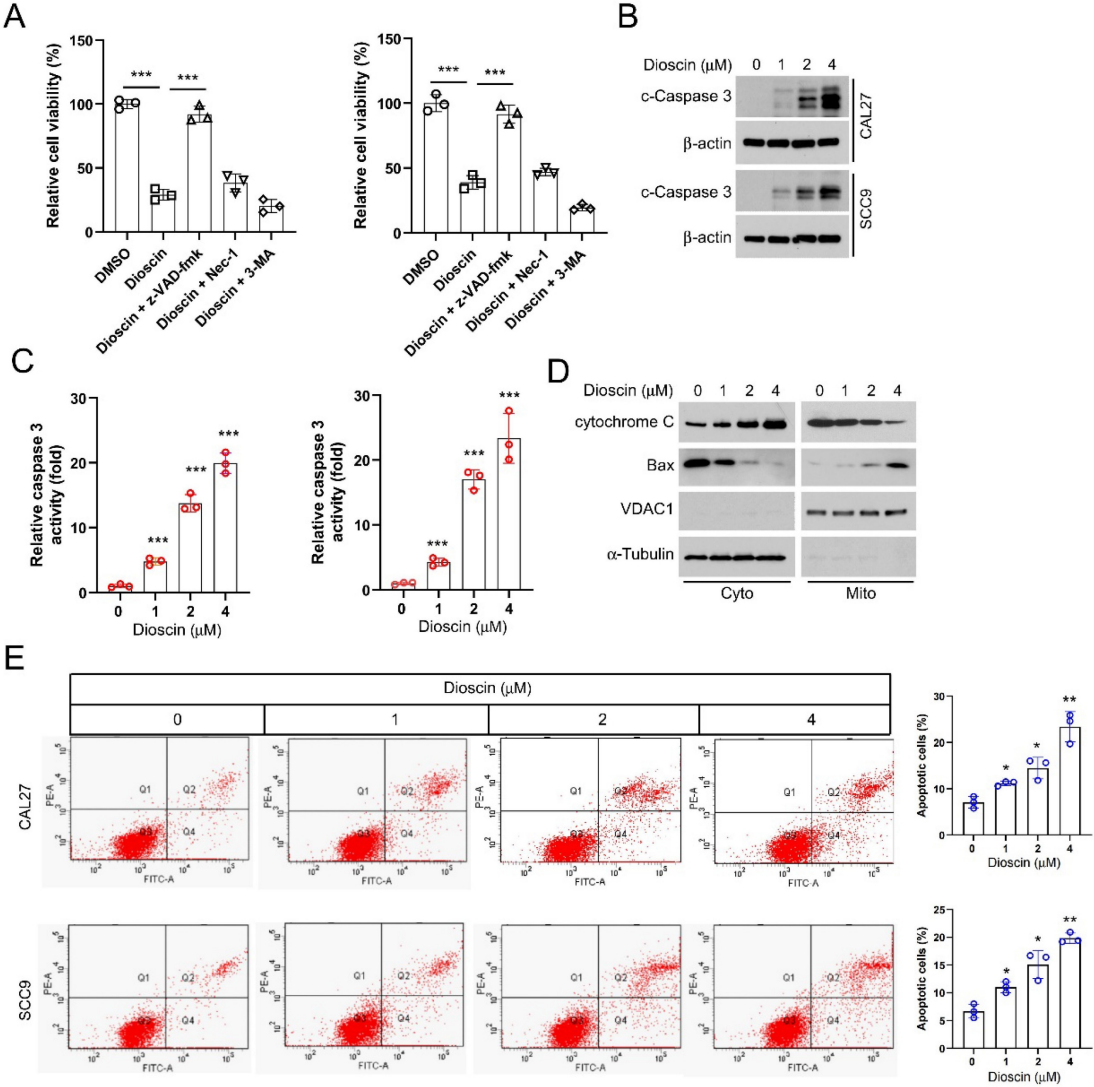
Dioscin induces intrinsic apoptosis in OSCC. A. CAL27 and SCC9 cells were pretreated with z-VAD-FMK, 3-MA, or Nec-1 for 4 h, followed by treatment with Diosicn for 48 h. MTS was performed to detect cell viability. B and C. CAL27 and SCC9 cells were treated with Dioscin (0/1/2/4 μM) for 48 h. WB assay was performed to detect the expression of cleaved caspase 3 (B); Caspase 3 activity was checked using the Caspase 3 assay kit (C). D. CAL27 cells were treated with Dioscin (0/1/2/4 μM) for 48 h. Subcellular fractions were isolated for detection of cytochrome c and Bax expression using the WB assay. E. Apoptosis was detected by flow cytometry in SCC9 and CAL 27 cells treated with different concentrations of Diosin.

To determine how Dioscin exerts an inhibitory effect on OSCC cells, we investigated whether Dioscin induces cell death. CAL27, SCC9, and SCC25 cells were treated with Dioscin in combination with three cell death inhibitors (the apoptosis inhibitor z-VAD-fmk, the necroptosis inhibitor Necrostatin-1, and the autophagy inhibitor 3-MA) for 24 h. The results showed significant recovery of cell viability in OSCC cells treated with Dioscin when co-incubated with the apoptosis inhibitor z-VAD-fmk. However, co-incubation with either the necrosis inhibitor Necrostatin-1 or the autophagy inhibitor 3-MA did not result in significant changes in cell viability (Figure 2A). Next, the protein level and enzymatic activity of cleaved-caspase 3 in Dioscin-treated SCC9 and CAL27 cells were further determined, and the data showed that its expression and activity increased, correlating with higher Dioscin concentrations (Figure 2B and 2C). Furthermore, after isolating subcellular fractions of SCC9 and CAL27 cell lysates, we observed that the cytochrome C protein levels were increased in the cytoplasm while decreasing in the mitochondria. The levels of Bax proteins were increased in the mitochondria but decreased in the cytoplasm following treatment with Dioscin (Figure 2D). Moreover, flow cytometry results also showed that Dioscin enhanced apoptosis in SCC9 and CAL27 cells in a dose-dependent manner (Figure 2E). All of these data suggest that Dioscin exerts antitumor effects by activating intrinsic apoptotic signaling pathways in OSCC cells.

### Diosicn induces OSCC cell apoptosis through BMI1-mediated Noxa upregulation

**Figure 3:**
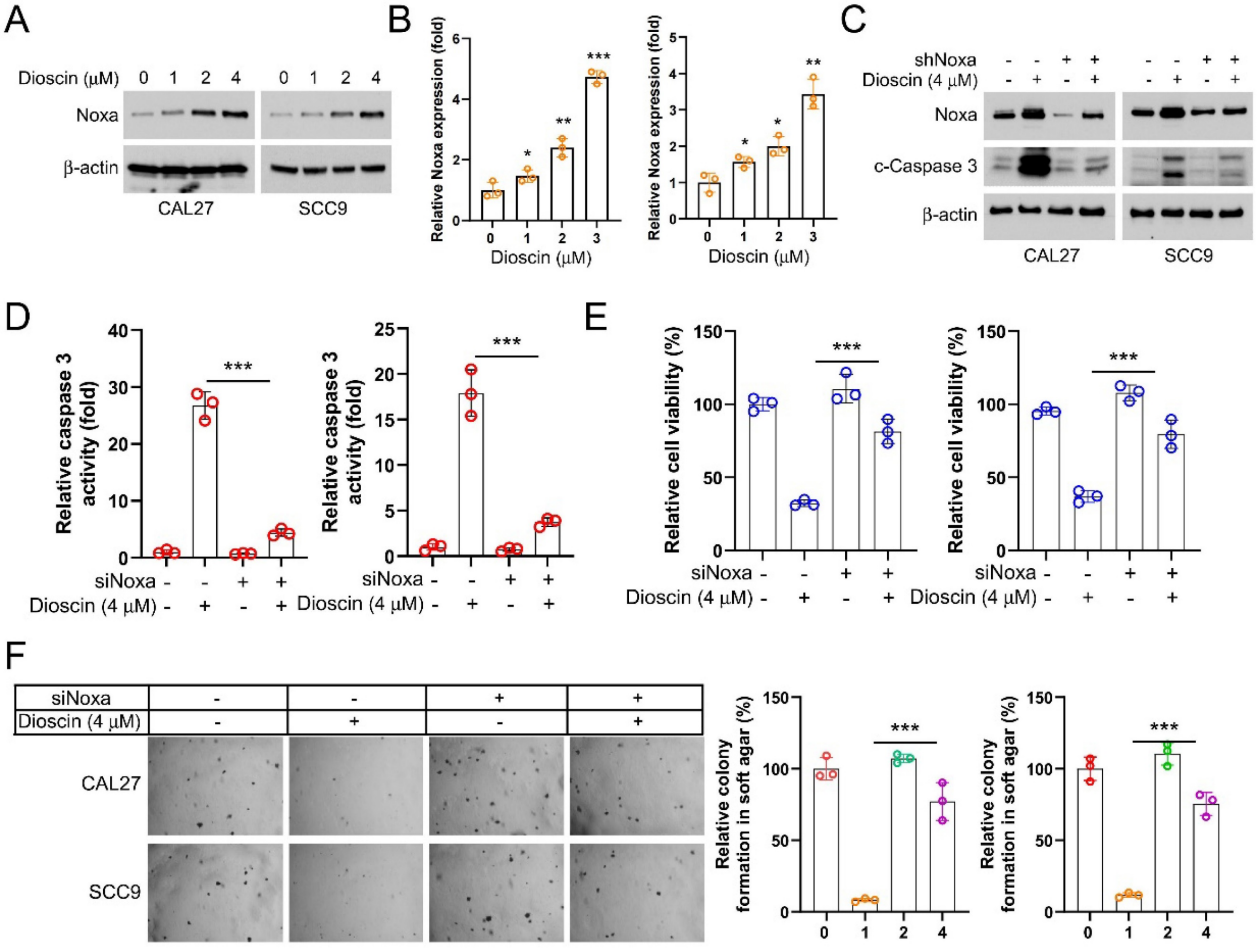
Diosin increases Noxa expression to induce apoptosis in OSCC cells. A. WB analysis of Noxa expression in Dioscin-treated CAL27 and SCC9 cells. B. qRT-PCR test analysis of Noxa expression in Dioscin-treated CAL27 and SCC9 cells C. WB analysis of Noxa and cleaved caspase 3 expression of CAL27 and SCC9 cells expressing shCtrl or shNoxa treated with Diosicn. D-F. siNoxa was transfected into CAL27 and SCC9 cells, followed by Diosicn treatment. Caspase 3 assay kit was used to detect caspase 3 activity (D), MTS assay for cell viability (E), and soft agar assay was used to detect anchorage-independent growth capacity (F).

In order to explore the potential molecular mechanisms of the antitumor effect of Dioscin, we detected Noxa expression in CAL27 and SCC9 cells. The WB data showed that the protein level of Noxa was markedly upregulated in OSCC cells treated with Dioscin (Figure 3A and 3B). In addition, Noxa knockdown decreased both the protein level and activity of cleaved-caspase3 in OSCC cells even in the presence of Dioscin exposure (Figure 3C and 3D). It also counteracted the Dioscin-induced inhibition of cell viability and colony formation (Figure 3E and 3F). These results suggest that Dioscin promotes apoptosis activation in OSCC cells by upregulating Noxa.

**Figure 4:**
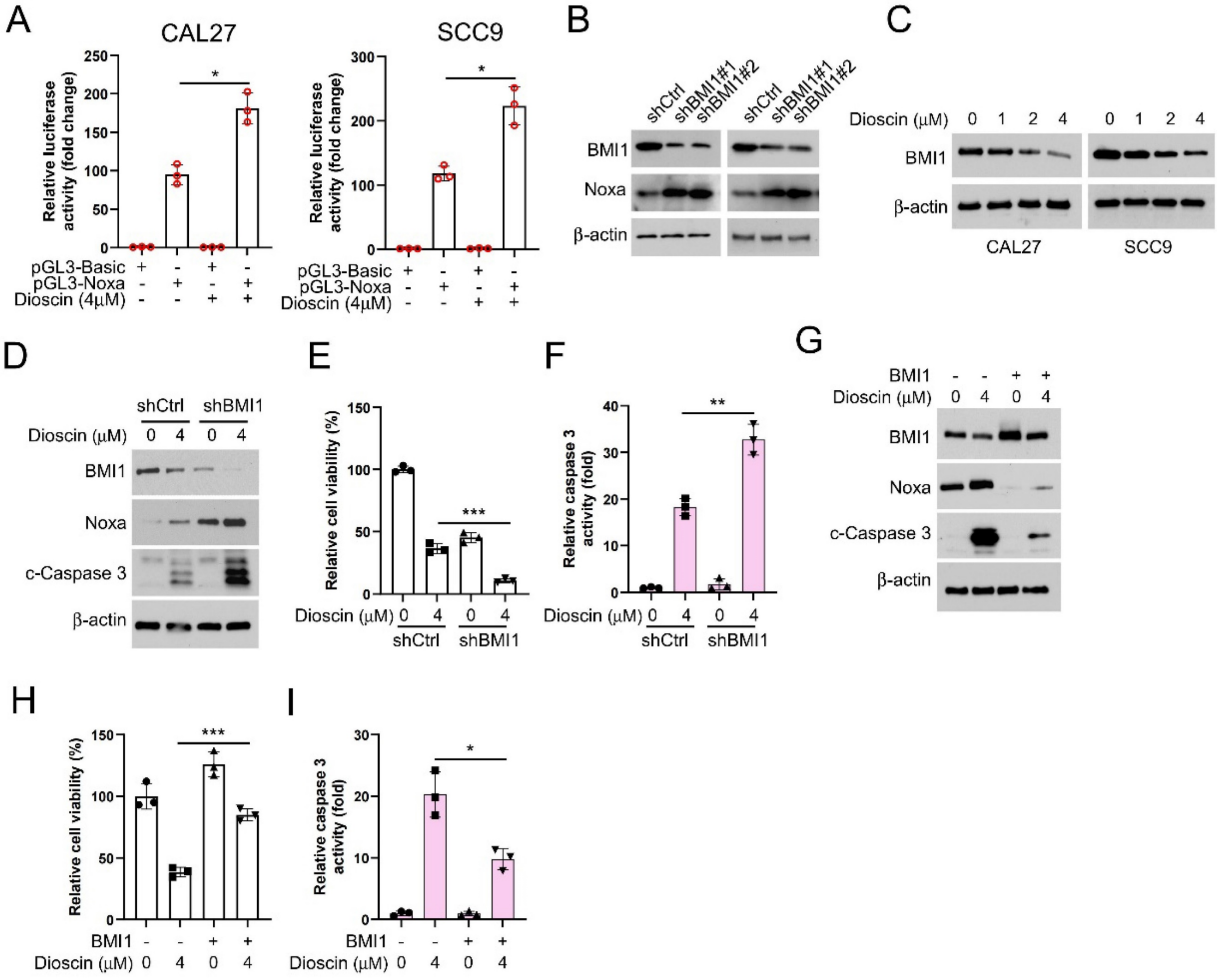
Diosicn induces apoptosis in OSCC cells through BMI1-mediated Noxa upregulation. A. CAL27 and SCC9 cells were transfected with a Noxa promoter reporter plasmid (pGL3-Noxa-N1) or pGL3-alkaline vector (pGL3-Basic) and then exposed to Dioscin for 48 h. Firefly luciferase readings were normalized to sea kidney luciferase to correct for transfection efficiency. Noxa promoter-driven luciferase activity was expressed as a fold induction of pGL3-alkaline vector activity. B. WB analysis was performed on BMI1 knockdown CAL27 and SCC9 cells. C. WB analysis was performed on Dioscin (0/1/2/4 µM) treated CAL27 and SCC9 cells. D-E. pLKO.1-shBMI1 was transfected into CAL27 cells and treated with Dioscin for 48 h, WB assay for BMI1, Noxa and c-Caspase 3 protein content in whole-cell lysates (D), MTS assay for cell viability (E), and Caspase 3 Assay Kit was used to examine caspase 3 activity (F). G-I. pT3-EF1a-BMI1 was transfected into CAL27 cells and treated with Dioscin for 48 h, WB assay for levels of BMI1, Noxa, and c-Caspase 3 proteins in whole cell lysates (G), MTS assay for cell viability (H), and Caspase 3 assay kit for detection of caspase 3 activity (I).

Next, we employed the pGL3-Noxa reporter gene, where the luciferase reporter gene is fused to the Noxa promoter, enabling luciferase expression under the control of the Noxa promoter. This allowed us to investigate how Dioscin affects the upregulation of the Noxa expression. The results showed that in the presence of Dioscin, luciferase expression was significantly increased in CAL27 and SCC9 cells transfected with pGL3-Noxa (Figure 4A), suggesting that the upregulation of Noxa induced by Dioscin is related to the promotion of Noxa transcription. Previous study has reported that BMI1 controls the Noxa gene, thus affecting the survival of memory CD4^+^ T cell^43^. Therefore, we sought to elucidate the relationship between BMI1 and Noxa in OSCC cells. We initially investigated the expression regulatory relationship between BMI1 and Noxa in OSCC cells to validate this hypothesis. As shown in Figure 4B, the expression of Noxa was upregulated under BMI1 knockdown. In addition, we found a gradual decrease in BMI1 expression level with increasing concentrations of Dioscin (Figure 4C). As expected, in BMI1-knockdown cells treated with Dioscin, the expression of Noxa and cleaved-caspase 3 was further reduced (Figure 4D), cell viability was more obviously inhibited (Figure 4E), and the activity of caspase 3 was further significantly increased (Figure 4F). Conversely, overexpression of BMI1 reversed the Dioscin-induced upregulation of Noxa and cleaved-caspase 3 expression (Figure 4G), attenuated Dioscin-induced inhibition of cell viability (Figure 4H) and activation of caspase 3 (Figure 4I). These results suggest that BMI1 is involved in mediating the upregualtion of Noxa induced by Dioscin in OSCC cells.

### β-TrCP-mediated ubiquitination is required for Dioscin-induced BMI1 degradation

**Figure 5:**
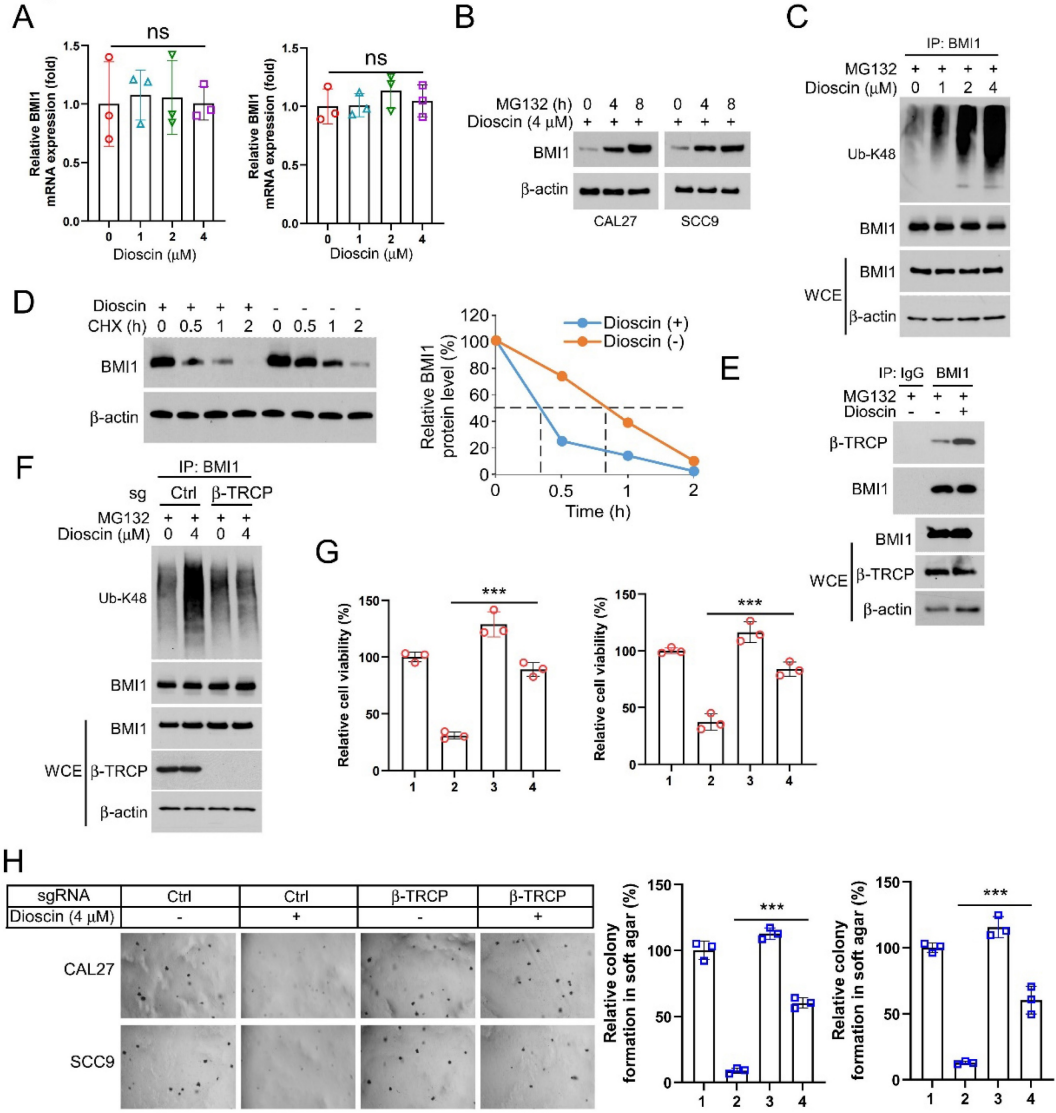
Dioscin induces BMI1 ubiquitination A. After Dioscin (4 μM) treatment, the mRNA level of BMI1 was measured using RT-qPCR assay. B. CAL27 and SCC9 cells were treated with Dioscin (4 μM) for 48 h and then treated with MG132 (20 μM) for 0/4/8 h, and the WB assay was performed to detect the protein levels of BMI1. C. CAL27 cells were treated with Dioscin (0/1/2/4 μM) for 48 h in combination with MG132 (20 μM) for 8 h. WCEs were prepared and analyzed for ubiquitination of BMI1 using ubiquitin antibody. D. CAL27 cells were treated with Dioscin for 48 h and incubated with CHX for different time points. WB analysis was performed to detect BMI1 expression. E. WCEs were collected for Co-IP assay in CAL27 cells treated with or without Dioscin (4 μM) for 48 h and then incubated with MG132 (4μM) for 8 h. F. CAL27 cells expressing sgCtrl or sgβ-TrCP were treated with Dioscin (4μM) for 48 h, and incubated with MG132 (20μM) for 6h. WB was performed to detect BMI1 ubiquitination analysis. G and H. CAL27 and SCC9 cells expressing sgCtrl or sgβ-TrCP were treated with Dioscin (4μM) for 48 h. MTS was performed to detect cell viability (G), soft agar assay was used to detect anchorage-independent growth capacity (H).

To elucidate how Dioscin reduces BMI1 expression at the intrinsic level, we first examined the mRNA level of BMI1 within CAL27 and SCC9 cells following Dioscin treatment, and the qRT-PCR results showed that there was no significant difference in the mRNA level between Dioscin-treated and control groups (Figure 5A), suggesting that Dioscin-induced downregulation of BMI1 expression is not through regulation of its transcriptional process. Next, we treated CAL27 and SCC9 cells with the proteasome inhibitor MG132. Surprisingly, WB results showed that co-incubation with MG132 restored the expression of BMI1 after treatment with Dioscin in a time-dependent manner (Figure 5B). Further study of ubiquitination experiments revealed that the ubiquitination level of BMI1 is noticeably upregulated with increasing concentration of Dioscin (Figure 5C). The cycloheximide chase assay demonstrated that the half-life of BMI1 was shortened under the condition of Dioscin (Figure 5D), suggesting that post-translational ubiquitination modification of BMI1 is closely related to its downregulation. β-TrCP functions as a substrate recognition receptor in the SCF E3 ubiquitin ligase complex. Its role is pivotal in identifying particular protein substrates and initiating their ubiquitination for subsequent degradation by the proteasome. This motif is pivotal in regulating the degradation of various proteins through the ubiquitin-proteasome pathway^44^. Sahasrabuddhe *et al.* found that^13^ β-TrCP regulates the turnover of BMI1 and its functions related to tumourigenesis, cellular senescence, and aging. To elucidate the interaction between β-TrCP and BMI1, Co- IP experiment was carried out and found that the interaction between β-TrCP and BMI1 was enhanced in Dioscin-treated CAL27 cells (Figure 5E). Subsequently, the results of ubiquitination experiments showed that knockdown of β-TrCP alleviated Dioscin-induced ubiquitination of BMI1 (Figure 5F), which resulted in stabilization of BMI1 expression and further disruption of the inhibitory effect of Dioscin on cell viability and colony formation ability (Figure 5G and 5H). This suggests that β-TrCP-mediated BMI1 Ub-K48 ubiquitination is necessary for Dioscin-induced BMI1 degradation.

### Dioscin inhibits tumor growth in xenograft models

**Figure 6:**
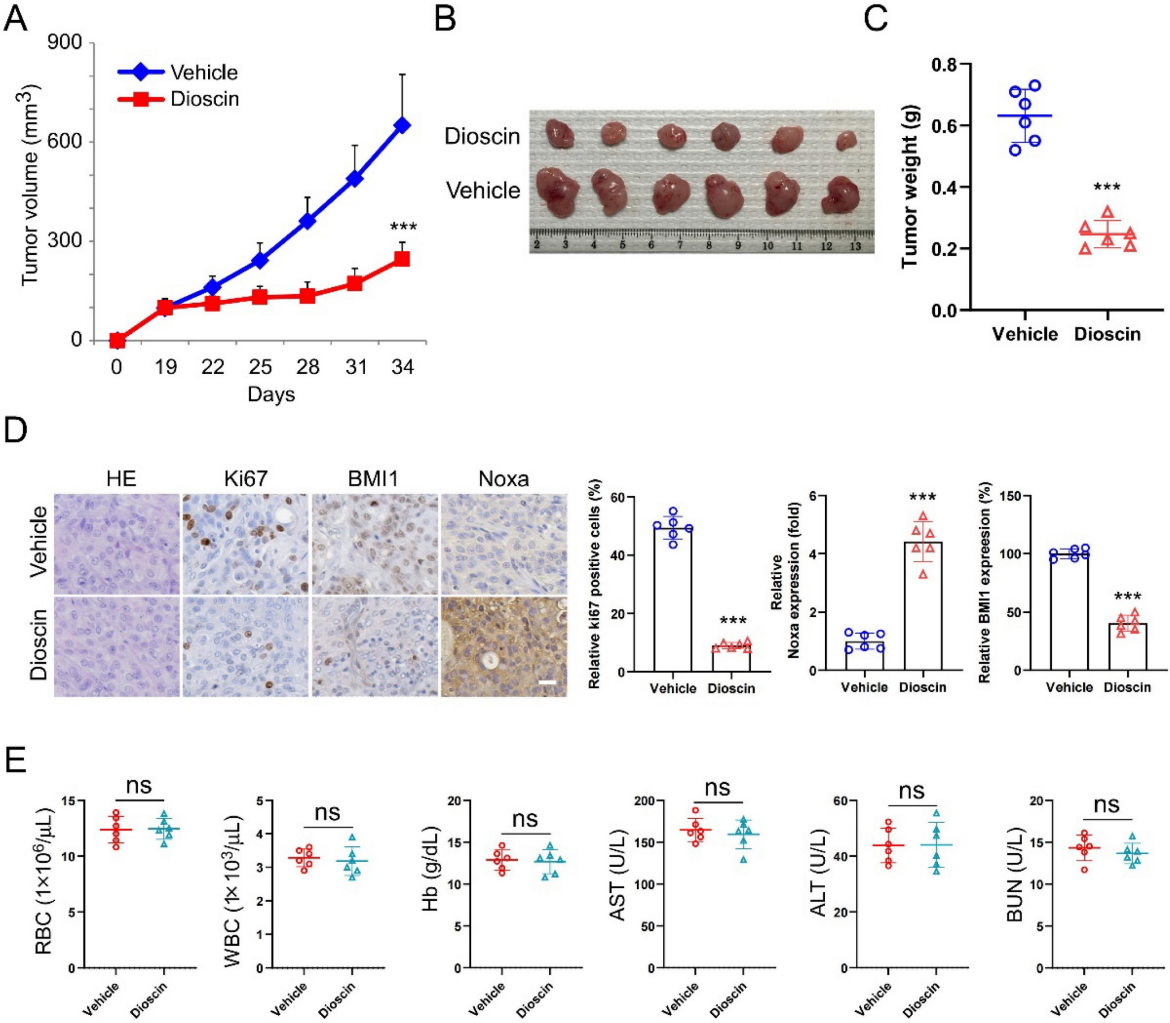
Dioscin inhibits tumor growth of OSCC cells *in vivo*. A-C. Volume (A), size (B) and weight (C) of CAL27-derived xenograft tumors treated with Vehicle or Dioscin. Scale bar, 1 cm. D. IHC staining assay for Ki67, BMI1, and Noxa expression of CAL27-derived xenograft tumor tissues treated with Vehicle or Dioscin. Scale bar, 25 μm. E. Blood assay of CAL27-derived xenograft treated with Vehicle or Dioscin. (ns, not statistically significant).

A xenograft mouse model was established to examine the potential of Dioscin to suppress tumor growth in living organisms. The study results indicated that Dioscin effectively suppressed the growth of CAL27 cells *in vivo*, as shown by the marked reduction of tumor volume and weight compared with the group treated with the vehicle (Figure 6A-C). Furthermore, the results of IHC labeling demonstrated that the number of Ki67-positive cells and the expression levels of BMI1 and Noxa were significantly decreased in the CAL27-xenograft model administrated Dioscin (Figure 6D). Notably, The levels of red blood cells (RBC), white blood cells (WBC), hemoglobin (Hb), aspartate aminotransferase (AST), alanine aminotransferase (ALT), and blood urea nitrogen (BUN) were not influenced by the administration of Dioscin, in comparison to the group treated with the vehicle (Figure 6E). These data suggest that Dioscin is a promising antitumor agent with good tolerance and can effectively inhibit the proliferation of OSCC cells in xenograft models.

## Discussion

Oral squamous cell carcinoma (OSCC) originates from the epithelial tissues of the oral mucosa and is among the most prevalent malignant tumors of the head and neck region. It is characterized by a high degree of malignancy, recurrence rates, lymph node metastasis, and generally poor prognosis^45^. Apoptosis is characterized by two primary pathways: the mitochondria-dependent (intrinsic) pathway and the death receptor-dependent (extrinsic) pathway^46^. The intrinsic pathway, mediated by Bax/Bak proteins, entails the release of cytochrome C^47^. In OSCC cells, apoptotic pathways are frequently inhibited.

Multiple natural products have strong apoptosis-inducing effects on tumors with minimal impact on normal cells. Dioscin, an active compound in medicinal plants such as Dioscoreaceae, Liliaceae, Vitis vinifera, Rosaceae, and other plants, exhibits various biological activities, including antioxidant, anti-inflammatory, and antitumor properties^23^. Currently, Dioscin is widely used in clinical practice in cardiovascular diseases, diabetes mellitus, and goiter-related diseases^26^, ^48^, ^49^. Several studies have indicated that Dioscin is also a multi-target drug for treating tumors^50^-^52^. Our study demonstrated that Dioscin activated apoptosis signaling by changing the intracellular distribution of cytochrome C and Bax in OSCC cells (Figure 2D), suggesting that Dioscin exerts antitumor cytotoxic effects by activating the intrinsic apoptosis pathway.

Noxa is a member of the BCL-2 family, which induces intrinsic apoptosis in various cancer cells by targeting BCL-1 ubiquitination to induce cell death^4^ and promoting Bax/Bak activation to exert its pro-apoptotic function^6^. In OSCC, Noxa is linked to the apoptotic process. Pristimerin, for example, induces apoptosis in OSCC cells (CAL27 and SCC25) by inhibiting Noxa expression^53^. Our data confirm that Dioscin regulates Noxa transcription and affects Noxa expression, thereby exerting anti-tumor effects in OSCC cells.

BMI1 is an intracellular oncoprotein that is implicated in the initiation and progression of tumor-initiating cells across various cancer types. It plays a crucial role in carcinogenesis and cancer progression. There was a statistically significant correlation between defective BMI1 immunoexpression and poor prognosis in patients with oral squamous cell carcinoma^22^. Additionally, studies^54^, ^55^ have indicated that BMI1 is highly expressed in oral pre-cancerous lesions such as erythema and lichen planus, and this high expression is associated with their malignant transformation, suggesting that BMI1 may play a significant role in the evolution of oral squamous cell carcinoma and the transition of pre-cancerous lesions into malignancies. BMI1 exerts its protumor effects through various downstream activities. For example, BMI1 promotes EMT in mammary epithelial cells and decreases their sensitivity to the chemotherapeutic drug doxorubicin^56^. Previous investigation has indicated the presence of a complex relationship between BMI1 and Noxa in immune cells^43^. However, this specific interaction has received limited attention in the field of oncology. In this study, we found that the interaction of BMI1 with Noxa was also present in OSCC cells and that the effect was amplified by Dioscin treatment.

β-TrCP is a substrate recognition receptor for the SCF E3 ubiquitin ligase complex responsible for recognizing specific protein substrates and initiating their ubiquitination for degradation^44^. Evidence demonstrates that in breast cancer, the overexpression of β-TRCP can result in reduced production of BMI1. Conversely, the knockdown of β-TRCP also leads to a reduction in protein hydrolysis of BMI1^13^. This finding offers a fresh perspective for our subsequent research. Our findings revealed that increasing Dioscin significantly raised BMI1 ubiquitination levels (Figure 5C). Knockdown of β-TrCP moderated Dioscin-induced BMI1 ubiquitination, which led to stabilization of BMI1 expression and disruption of the inhibitory effect of Dioscin on cell viability and colony formation ability (Figure 5F-H). These results suggest that Dioscin down-regulates BMI1 by promoting ubiquitination-linked processes mediated by β-TrCP, which regulates Noxa expression and ultimately induces apoptosis in OSCC cells. In xenograft model studies, it has been further demonstrated that Dioscin inhibits OSCC tumor growth *in vivo* without significant cytotoxic effects.

Overall, our study provides valuable insight into the potential use of Dioscin as a treatment for OSCC by targeting the BMI1-Noxa axis. This novel antitumor mechanism offers promising opportunities to develop new therapeutic strategies for OSCC.

## Funding

This work was supported by the Natural Science Foundation of Changsha (kq2208458), the Department of Science and Technology of Hunan Province (2024JJ9519), the Key Project of Hunan Provincial Department of Education (22A0249), the Natural Science Foundation of Hunan Province (2022J30630).
